# Evaluating postoperative recovery in uniportal versus needlescopic video-assisted thoracoscopic surgery for primary spontaneous pneumothorax: a comparable study

**DOI:** 10.3389/fsurg.2024.1356989

**Published:** 2024-02-28

**Authors:** Jen-Hao Chuang, Hsao-Hsun Hsu, Mong-Wei Lin, Pei-Ming Huang, Shuenn-Wen Kuo, Ke-Cheng Chen, Jin-Shing Chen

**Affiliations:** ^1^ Department of Surgical Oncology, National Taiwan University Cancer Center, National Taiwan University College of Medicine, Taipei, Taiwan; ^2^ Department of Surgery, National Taiwan University Hospital and National Taiwan University College of Medicine, Taipei, Taiwan

**Keywords:** primary spontaneous pneumothorax (PSP), video-assisted thoracoscopic surgery (VATS), pneumothorax, needlescopic, uniportal VATS (U-VATS)

## Abstract

**Objectives:**

Primary spontaneous pneumothorax (PSP) is a common disease in young and thin male. Operation has been regarded as definitive treatment for it. However, the operative methods for those patients are under dispute. This study aims to directly compare the outcomes of uniportal VATS vs. needlescopic VATS in the treatment of PSP, focusing on postoperative pain and safety outcomes.

**Methods:**

From July 2013 to December 2017, the patients who underwent video-assisted thoracic surgery for pneumothorax in National Taiwan University Hospital were retrospectively collected. The preoperative condition, surgical results, and postoperative outcomes was analyzed.

**Results:**

There were 60 patients undergoing needlescopic VATS and 91 undergoing uniportal VATS during the study period. There was no significant difference between the patients who underwent needlescopic VATS and those who underwent uniportal VATS in their demographic and clinical characteristics. The post-operative pain score was significantly lower in the uniportal VATS group compared to the needlescopic VATS group at day 1 (2.65 ± 1.59 vs. 1.74 ± 1.35, *p* = 0.001).

**Conclusion:**

Uniportal VATS offers an effective, safe alternative for PSP treatment, with benefits including reduced post-operative pain. Our findings support the use of uniportal VATS, supplemented by a wound protector, as a viable option for PSP patients.

## Introduction

Pneumothorax is defined as the presence of the air in the pleural cavity. Primary spontaneous pneumothorax (PSP) is more common in young, tall, lean men (1). After the first episode, the estimated recurrence rate is 32%, with a range from 17% to 54% (2, 3). The optimal management of PSP has been an issue for discussion for years. The American College of Chest physicians (ACCP) and the British Society of Thoracic Surgeons (BSTS) (4) have published their own guidelines for the management of PSP. Both reports stated that PSP should be treated surgically after the first recurrence, performing a thoracoscopic bullectomy associated with a procedure for inducing pleural adhesions. Therefore, the surgical approach is considered the best treatment to minimize the risk of recurrence in patients who experienced PSP.

Needlescopic equipment and instruments have been applied to thoracic procedures (5). The initial reports suggested that needlescopic VATS was feasible and resulted in better cosmesis and less postoperative pain for PSP patients. In contrast, uniportal VATS technique has shown to be safe and efficient not only for pulmonary resections and biopsies but also for lobectomy (6). Besides, uniportal VATS could reduce patients' postoperative pain and paraesthesia and improve patients’ satisfaction, concluding that the uniportal VATS technique is a safe, feasible and effective treatment for PSP (7). However, the comparison between these two methods lacks. In this study, we aim to provide a comprehensive comparison of uniportal vs. needlescopic video-assisted thoracoscopic surgery in the management of primary spontaneous pneumothorax, focusing on surgical outcomes, postoperative pain, and patient satisfaction.

## Materials and methods

### Study design and patients

The medical records of all patients of PSP, and who underwent VATS for treatment of it at National Taiwan University Hospital, a 3,200-bed tertiary medical center, during the period from July 2013 to December 2017 were retrospectively collected. The data included patients in needlescopic and uniportal VATS groups. The preoperative condition, surgical results, and postoperative short-term outcomes was analyzed. The Research Ethics Committee of National Taiwan University Hospital approved this study (approval number: 201803043RINA) and waived the requirement for informed consent due to the study's retrospective nature. It was conformed that all methods were performed in accordance with the relevant guidelines and regulations.

Eligibility for inclusion required a confirmed diagnosis of PSP via chest radiography or computed tomography (CT). The choice between uniportal and needlescopic VATS was made based on a combination of patient-specific factors, including the size and location of the pneumothorax, previous surgical history, and patient preference, after thorough discussion of the potential risks and benefits of each approach.

### Surgical technique

#### Needlescopic VATS

Under general anesthesia with single lung ventilation, the patients were turned into the lateral decubitus position. Following disinfection, a 12 mm port was made at the sixth or seventh intercostal space for camera insertion. To facilitate the use of needlescopic instruments, three small skin punctures were created, with the inferior mini-port positioned at the seventh or eighth intercostal space along the mid-axillary line, directly below the level of the chest tube wound. The two superior mini-ports were located at the fourth and fifth intercostal space of the anterior and posterior axillary lines, respectively (5, 6).

Initially, the 10 mm telescope and two mini-endograspers were used to identify the blebs. When a bleb was identified, it was fixed by mini-endograsper at one of the superior mini-ports. A 3 mm 30 needlescope was introduced at the inferior mini-port to visualize the bleb. The 10-mm telescope was then withdrawn, and a 45 mm endoscopic linear stapler was introduced to resect the bleb. If no air leakage was identified, apical stapling was routinely performed at the most suspicious area. The surgical specimen was retrieved from the chest tube wound. The 10 mm telescope was inserted again to check the stapling line. Following the assessment for air leaks and bleeding, a chest tube ranging from 20 to 28 French was inserted at the apex of the pleural cavity through the inferior mini-port, after which the wound was closed in layers (5, 6).

#### Uniportal VATS

After preparing the surgical site, a single incision of 2.5 cm was made in the fifth intercostal space along the mid/posterior axillary line, utilizing a wound protector to facilitate access without a trocar. Once lung deflated, a 5-mm, rigid endoscope with 30-degree viewing angle was then inserted to the pleural space to confirm the lesion. An endoscopic grasping instrument and endoscopic stapler were inserted through the same window. Bullectomy and/or blebectomy were operated under direct thoracoscopic vision with endostaplers. Then we completed mechanical pleurodesis by pleural abrasion with scratch pad. After checking air-leak and bleeding, a 20–28 French chest tube was placed in the apex of pleural cavity and the wound was closed in layers (7).

### Data collection and analysis

In our analysis, we focused on evaluating several key outcomes to assess the effectiveness and safety of uniportal vs. needlescopic VATS for PSP treatment. Specifically, we measured postoperative pain scores (using a 0–10 visual analog scale), duration of hospital stay (in days), incidence of postoperative complications, and rate of pneumothorax recurrence within a six-month follow-up period. All continuous values were presented as mean ± standard deviation. Demographic information (age, gender, BMI) and clinical data (size of pneumothorax, previous episodes of PSP, operative time) of the different surgical groups were compared using the χ^2^ test or Fisher's exact test for categorical variables, and the Student's *t*-test or Mann–Whitney *U*-test for continuous variables, as appropriate. All of the analyses were performed with the statistical software package SPSS (SPSS, Inc., Chicago, IL, USA).

## Results

Of the 151 subjects included in this study, 60 underwent needlescopic VATS and 91 underwent uniportal VATS. There was no significant difference between the patients who underwent needlescopic VATS and those who underwent uniportal VATS in demographic and clinical characteristics (Table 1). One patient in the uniportal VATS group had bilateral pneumothoracex, and thus underwent left upper lobe and right upper lobe pulmonary wedge resection by changing the position. No cases were converted with other surgical methods during operation.

**Table 1:** Demographic and clinical characteristics of study groups of PSP.

	Needlescopic VATS (*n* = 60)	Uniportal VATS (*n* = 91)	*p*-value
Age, mean ± SD, years	20.85 ± 5.90	23.53 ± 8.23	0.03
Male	50	74	
Female	10	17	
BMI	19.22 ± 2.40	19.09 ± 2.46	
Smoking	7	8	0.56
Pneumothorax site			NS
Right	22	39	
Left	37	51	
Bilateral	0	1	
Pre-OP intervention	23	31	NS

BMI, body mass index.

There was no statistically significant difference in surgical outcomes between needlescopic and uniportal VATS (Table 2). All four cases of persistent air leakage, defined as air leakage that continues for more than 5–7 days after surgery, were successfully treated with chemical pleurodesis (8). Chemical pleurodesis was performed using OK-432 (Picibanil), a sclerosing agent, injected through the chest tube to promote pleural adhesion and cease the air leak, facilitating the removal of the chest tube (9). One case of massive pleural effusion was treated successfully after prolonged chest tube drainage. The post-operative pain score was significantly lower in the uniportal VATS group compared to the needlescopic VATS group at day 1 (2.65 ± 1.59 vs. 1.74 ± 1.35, *p* = 0.001). Although there was no significant difference in the use of additional intramuscular analgesia in the two groups both on post-operative 3 days and discharge, the dose was still lower in the uniportal VATS group (0.78 ± 0.94 vs. 0.69 ± 0.98, *p* = 0.545). Both groups had three cases of recurrent pneumothorax in the during the follow-up period. All cases were treated by re-do VATS that led to successful re-expansion of the lung. Additional treatment was not indicated as there was no further air leakage.

**Table 2:** Surgical outcomes between needlescopic VATS and uniportal VATS for PSP.

	Needlescopic VATS (*n* = 60)	Uniportal VATS (*n* = 91)	*p*-value
Operation time, minutes	55.80 ± 21.93	53.49 ± 20.40	0.51
Hospital stay, days	3.6 ± 1.66	3.25 ± 0.85	0.95
Chest tube drainage days	2.7 ± 1.4	2.35 ± 0.92	0.17
Complications, no. (%)			
Hemothorax	0	0	NS
Persistent air leak (chemical pleurodesis)	1	3	
Massive pleural effusion	0	1	
Pain score			
Day 0	2.65 ± 1.59	1.74 ± 1.35	0.001
Day 1	1.97 ± 1.20	1.70 ± 0.98	0.16
Day 2	1.54 ± 0.89	1.3 ± 0.84	0.12
Intravenous analgesics	0.78 ± 0.94	0.69 ± 0.98	0.55
Recurrence	3	3	0.78

## Discussion

Pneumothorax exists with a disease spectrum, ranging from devastating emergency to asymptomatic minor abnormality (8–11). There are lots of innovative treatments proposed in recent decades (1–5, 7, 12–16). Most thoracic surgeons worldwide are well trained to perform VATS bullectomy on patients with PSP, but many inconsistencies have been found in terms of how they perform VATS. Different methods have their individual risks. In most cases, the disease course is not so lethal that recurrence is the main problem. While it is not difficult to drain air from the body using a drain and chest bottle, the major challenge is to prevent reoccurrence (17–19). Therefore, the operation target is resuming complete expansion of the lung and prevention of recurrent pneumothorax. Clinically, indications for surgery include ipsilateral or contralateral recurrent episodes; persistent air leaks from chest tube (>5–7 days) or radiological persistence of pneumothorax after the same period; bilateral pneumothorax; concurrent hemothorax; risk occupation and pregnancy (4). Thus, pleurectomy or pleural abrasion by VATS is better tolerated but has a higher recurrence rate (approximately 5%). Our previous study also showed that needlescopic VATS is feasible in the treatment of PSP (3).

On the other hand, uniportal VATS has been proposed for innovative minimally invasive approach for surgical management in PSP for many years. In this study, we found the feasibility, complications, and short-term outcomes of needlescopic VATS with those of uniportal VATS procedures are quite similar. Uniportal VATS has the advantageous post-operative pain score while others parameters seem equal and non-inferior. This reduction in pain could potentially facilitate quicker recovery periods, decrease the need for analgesics, and improve overall patient experience post-surgery.

Moreover, the similarity in short-term outcomes between the two techniques suggests that the choice of surgical approach can be tailored to the surgeon's expertise and patient preferences without compromising the effectiveness of PSP treatment.

In addressing the management of primary spontaneous pneumothorax (PSP), our study contributes to a nuanced understanding of the comparative effectiveness of uniportal vs. needlescopic VATS. The epidemiological profile and treatment landscape of PSP have evolved, with Hallifax et al. highlighting a significant increase in hospital admissions for PSP over a nearly five-decade span, underscoring the importance of effective management strategies for this condition (20). This trend necessitates a reevaluation of surgical approaches to optimize patient outcomes and healthcare resource utilization.

Our findings, which indicate no significant difference in post-operative outcomes between the two surgical techniques, resonate with the ongoing debate regarding the optimal management of PSP. The study by Brown et al. provides a pertinent comparison, suggesting that conservative management of PSP may be noninferior to interventional approaches, with a lower risk of serious adverse events (21). This insight is crucial, as it frames our results within a broader context of seeking less invasive yet effective treatments for PSP, aligning with our observation of the minimal difference in efficacy between uniportal and needlescopic VATS.

Moreover, the exploration of ambulatory management by Bintcliffe et al. sheds light on alternative PSP treatment modalities that could further reduce hospitalization duration and associated healthcare costs, without compromising safety (22). Their findings support our study's implication that advancements in PSP management should not only focus on surgical innovation but also on enhancing patient recovery and system efficiencies.

In light of these studies, our research underscores the need for a multifaceted approach to PSP treatment that considers patient preferences, resource availability, and the potential benefits of newer, less invasive techniques. The gradual shift towards uniportal VATS in our practice, post-2017, reflects an adaptation to these evolving treatment paradigms, aiming to balance surgical efficacy with patient-centered care outcomes.

Despite these promising findings, it is essential to acknowledge our study's limitations, including its retrospective design, which may introduce selection bias and limit the generalizability of the results. Future prospective studies with larger sample sizes are needed to confirm these findings and explore long-term outcomes, such as recurrence rates and quality of life post-surgery.

## Conclusion

Our comparative analysis of needlescopic VATS and uniportal VATS for PSP management has demonstrated that uniportal VATS is an effective, safe, and potentially more patient-friendly option due to its association with lower post-operative pain. The findings support the use of uniportal VATS as a viable surgical alternative for treating PSP, offering benefits that align with the goals of modern thoracic surgery: minimizing invasiveness, enhancing patient comfort, and ensuring optimal clinical outcomes. As the field of minimally invasive thoracic surgery continues to evolve, ongoing research and innovation will be crucial in refining surgical techniques and improving patient care in PSP management.

## Data Availability

The raw data supporting the conclusions of this article will be made available by the authors, without undue reservation.

## References

[1] Tschopp JM Bintcliffe O Astoul P Canalis E Driesen P Janssen J ERS task force statement: diagnosis and treatment of primary spontaneous pneumothorax. Eur Respir J. (2015) 46(2):321–35. 10.1183/09031936.00219214 26113675

[2] Massongo M Leroy S Scherpereel A Vaniet F Dhalluin X Chahine B Outpatient management of primary spontaneous pneumothorax: a prospective study. Eur Respir J. (2014) 43(2):582–90. 10.1183/09031936.00179112 23766331

[3] Chen JS Chan WK Tsai KT Hsu HH Lin CY Yuan A Simple aspiration and drainage and intrapleural minocycline pleurodesis versus simple aspiration and drainage for the initial treatment of primary spontaneous pneumothorax: an open- label, parallel-group, prospective, randomised, controlled trial. Lancet. (2013) 381(9874):1277–82. 10.1016/S0140-6736(12)62170-9 23489754

[4] Henry M Arnold T Harvey J, Pleural Diseases Group, Standards of Care Committee, British Thoracic Society. BTS guidelines for the management of spontaneous pneumo- thorax. Thorax. (2003) 58(Suppl 2):ii39–52. 10.1136/thx.58.suppl_2.ii39 12728149 PMC1766020

[5] Chang YC Chen CW Huang SH Chen JS. Modified needlescopic video-assisted thoracic surgery for primary sponta-neous pneumothorax: the long-term effects of apical pleurectomy versus pleural abrasion. Surg Endosc. (2006) 20:757–62. 10.1007/s00464-005-0275-6 16437271

[6] Gonzalez-Rivas D Fieira E Delgado M Mendez L Fernandez R de la Torre M. Uniportal video-assisted thoracoscopic lobectomy. J Thorac Dis. (2013) 5(Suppl 3):S234–45. 10.3978/j.issn.2072-1439.2013.07.30 24040531 PMC3771611

[7] Berlanga LA Gigirey O. Uniportal video-assisted thoracic surgery for primary spontaneous pneumothorax using a single-incision laparoscopic surgery port: a feasible and safe procedure. Surg Endosc. (2011) 25(6):2044–7. 10.1007/s00464-010-1470-7 21136111

[8] Dugan KC Laxmanan B Murgu S Hogarth DK. Management of persistent air leaks. Chest. (2017) 152(2):417–23. 10.1016/j.chest.2017.02.020 28267436 PMC6026238

[9] How CH Hsu HH Chen JS. Chemical pleurodesis for spontaneous pneumothorax. J Formos Med Assoc. (2013) 112(12):749–55. 10.1016/j.jfma.2013.10.016 24268613

[10] Hyde B Hyde L. Spontaneous pneumothorax—contrast of the benign idiopathic and the tuberculous types. Ann Intern Med. (1950) 33:1373–7. 10.7326/0003-4819-33-6-1373 14790519

[11] Kjæergaard H. Spontaneous pneumothorax in the apparently healthy. Acta Med Scand Suppl. (1932) 43:1–159.

[12] Gossot D Galetta D Stern JB Debrosse D Caliandro R Girard P Results of thoracoscopic pleural abrasion for primary spontaneous pneumothorax. Surg Endosc. (2004) 18:466–71. 10.1007/s00464-003-9067-z 14752638

[13] Huh U Kim YD Cho JS IH Lee JG Lee JH. The effect of thoracoscopic pleurodesis in primary spontaneous pneumothorax: apical parietal pleurectomy versus pleural abrasion. Korean J Thorac Cardiovasc Surg. (2012) 45:316–9. 10.5090/kjtcs.2012.45.5.316 23130305 PMC3487015

[14] Moreno-Merino S Congregado M Gallardo G Jimenez-Merchan R Trivino A Cozar F Comparative study of talc poudrage versus pleural abrasion for the treatment of primary spontaneous pneumothorax. Interact Cardiovasc Thorac Surg. (2012) 15:81–5. 10.1093/icvts/ivs027 22514256 PMC3380967

[15] Chen JS Hsu HH Huang PM Kuo SW Lin MW Chang CC Thoracoscopic pleurodesis for primary spontaneous pneumothorax with high recurrence risk: a prospective randomized trial. Ann Surg. (2012) 255:440–5. 10.1097/SLA.0b013e31824723f4 22323011

[16] Park JS Han WS Kim HK Choi YS. Pleural abrasion for mechanical pleurodesis in surgery for primary spontaneous pneumothorax: is it effective? Surg Laparosc Endosc Percutan Tech. (2012) 22:62–4. 10.1097/SLE.0b013e31823cc61e 22318062

[17] Aihara K Handa T Nagai S Tanizawa K Watanabe K Harada Y Efficacy of blood-patch pleurodesis for secondary spontaneous pneumothorax in interstitial lung disease. Intern Med. (2011) 50:1157–62. 10.2169/internalmedicine.50.4645 21628929

[18] Rena O Massera F Papalia E Della Pona C Robustellini M Casadio C. Surgical pleurodesis for vanderschueren’s stage III primary spontaneous pneumothorax. Eur Respir J. (2008) 31:837–41. 10.1183/09031936.00140806 18057049

[19] Colt HG Russack V Chiu Y Konopka RG Chiles PG Pedersen CA A comparison of thoracoscopic talc insufflation, slurry, and mechanical abrasion pleurodesis. Chest. (1997) 111:442–8. 10.1378/chest.111.2.442 9041994

[20] Hallifax RJ Goldacre R Landray MJ Rahman NM Goldacre MJ. Trends in the incidence and recurrence of inpatient-treated spontaneous pneumothorax, 1968–2016. JAMA. (2018) 320(14):1471–80. 10.1001/jama.2018.14299 30304427 PMC6233798

[21] Hallifax RJ McKeown E Sivakumar P Fairbairn I Peter C Leitch A Ambulatory management of primary spontaneous pneumothorax: an open-label, randomised controlled trial. Lancet. (2020) 396(10243):39–49. 10.1016/S0140-6736(20)31043-6 32622394 PMC7607300

[22] Brown SGA Ball EL Perrin K Asha SE Braithwaite I Egerton-Warburton D Conservative versus interventional treatment for spontaneous pneumothorax. N Engl J Med. (2020) 382(5):405–15. 10.1056/NEJMoa1910775 31995686

